# The Communities First (ComFi) study: protocol for a prospective controlled quasi-experimental study to evaluate the impact of area-wide regeneration on mental health and social cohesion in deprived communities

**DOI:** 10.1136/bmjopen-2014-006530

**Published:** 2014-10-14

**Authors:** James White, Giles Greene, Frank Dunstan, Sarah Rodgers, Ronan A Lyons, Ioan Humphreys, Ann John, Chris Webster, Stephen Palmer, Eva Elliott, Ceri J Phillips, David Fone

**Affiliations:** 1 South East Wales Trials Unit (SEWTU), School of Medicine, Cardiff, UK; 2 Centre for the Development and Evaluation of Complex Interventions for Public Health Improvement (DECIPHer), School of Medicine, Cardiff, UK; 3 Institute of Primary Care & Public Health, Neuadd Meirionnydd, School of Medicine, Cardiff University, Cardiff, UK; 4 Farr Institute, College of Medicine, Swansea University, Swansea, UK; 5 Swansea Centre for Health Economics, College of Human and Health Sciences, Swansea University, Swansea, UK; 6 Faculty of Architecture, Department of Urban Planning and Design, The University of Hong Kong, Hong Kong, Hong Kong; 7 Cardiff Institute of Society, Health and Wellbeing (CISHeW), School of Social Sciences, Cardiff University, Cardiff, UK

**Keywords:** MENTAL HEALTH, PUBLIC HEALTH, EPIDEMIOLOGY

## Abstract

**Introduction:**

Recent systematic reviews have highlighted the dearth of evidence on the effectiveness of regeneration on health and health inequalities. ‘Communities First’ is an area-wide regeneration scheme to improve the lives of people living in the most deprived areas in Wales (UK). This study will evaluate the impact of Communities First on residents’ mental health and social cohesion.

**Methods and analysis:**

A prospective controlled quasi-experimental study of the association between residence in Communities First regeneration areas in Caerphilly county borough and change in mental health and social cohesion. The study population is the 4226 residents aged 18–74 years who responded to the Caerphilly Health and Social Needs Study in 2001 (before delivery) and 2008 (after delivery of Communities First). Data on the location, type and cost of Communities First interventions will be extracted from records collected by Caerphilly county borough council. The primary outcome is the change in mental health between 2001 and 2008. Secondary outcomes are changes: in common mental disorder case status (using survey and general practice data), social cohesion and mental health inequalities. Multilevel models will examine change in mental health and social cohesion between Communities First and control areas, adjusting for individual and household level confounding factors. Further models will examine the effects of (1) different types of intervention, (2) contamination across areas, (3) length of residence in a Communities First area, and (4) population migration. We will carry out a cost-consequences analysis to summarise the outcomes generated for participants, as well as service utilisation and utility gains.

**Ethics and dissemination:**

This study has had approval from the Information Governance Review Panel at Swansea University (Ref: 0266 CF). Findings will be disseminated through peer-review publications, international conferences, policy and practice partners in local and national government, and updates on our study website (http://medicine.cardiff.ac.uk/clinical-study/communities-first-regeneration-programme/).

**Strengths and limitations of this study**

- The study is a prospective controlled evaluation of a natural experiment which has detailed mental health data from an anonymously record-linked prospective cohort (eCATALyST) and general practice such that we will have detailed assessments on individual and household-level social, economic and health-based confounding factors.
- The study is sufficiently powered to detect an effect of the Communities First programme on mental health.
- Detailed data are available on the costs of the intervention and the use of health services, such that a cost consequences analysis will be carried out.

## Introduction

Recently there has been political appetite for large scale programmes to address the wider social, economic and environmental determinants of poor health through urban regeneration and neighbourhood renewal projects. It is estimated that in the over past 20 years £11 billion has been spent on these schemes in the UK.^1^ These regeneration schemes are typically designed to improve the likelihood of employment, education and social support within economically deprived communities, rather than to directly improve health. The interventions delivered in these schemes therefore include a broad range of regeneration activities to: (1) improve the built environment: increase access to public transport, create or maintain green space, (2) improve housing quality: provide free loft insulation, double glazing,^2^ ^3^ (3) reduce crime and promote safety: install street lighting, alarms, traffic calming and pedestrian zones, (4) reduce environmental stressors such as litter and noise, and (5) promote social support and contact between residents: by building or staffing youth clubs, providing sports equipment; setting up luncheon or widows clubs.^4^ Although there has been a number of systematic reviews examining on the health impacts of housing improvement,^5^ ^6^ and a few evaluations area-regeneration schemes on short-term changes in employment, education and income,^7^ ^8^ there is a dearth of evidence on the effectiveness of area-wide regeneration schemes on health outcomes.^1^

We identified three studies which have evaluated the impact of area-regeneration schemes on mental health.^9^ ^10^ An evaluation of the New Deal for Communities, delivered in deprived areas in England, found no difference in the change in mental health scores (2002–2008) between residents of New Deal areas and residents randomly sampled from non-contiguous comparator areas^9^; or participants in the Health Survey for England stratified according to levels of area deprivation.^10^ An evaluation of the Go Well regeneration programme, based in Glasgow reported a small improvement in mental health was associated with housing repairs and improvements, but no difference in mental health between residents living in intervention areas being demolished compared to residents from matched control areas.^11^

‘Communities First’ is a Welsh Assembly Government programme of area-wide regeneration delivered in the most deprived communities in Wales. Communities First has spent around £300 million up until 2012 (equivalent to an average of some £200 000 per community or around £55 per resident per annum).^12^ To date, evaluations of Communities First have included two reports based on process data which suggested the scheme was viewed positively by residents and may have had a beneficial effect on physical health^13^ ^14^; and another evaluation of the Communities First Support Network made recommendations on how best to support the Communities First programmes.^15^ A comparison across Wales between residents who did and did not live in a Communities First area using routine government data aggregated at a small area level suggested there was very little impact on levels of unemployment, unemployment benefit, educational achievement and crime. There was, however, no evaluation of Communities First on mental health.^14^

The proposed study exploits an opportunity to nest a prospective controlled quasi-experimental study to investigate the effects of Communities First within an electronic record-linked prospective cohort, the Caerphilly Health and Social Needs Electronic Cohort Study (eCATALYsT).^16^ We will collect information on the type, location and costs of Communities First interventions in Caerphilly which will be anonymously record-linked to eCATALyST study. The eCATALyST study collected data on mental health, social cohesion before and after the Communities First programme, from residents who did and did not reside in Communities First areas, as well as providing detailed assessments on changes in household and individual-level socioeconomic status. We will also collect mental health data from general practice to triangulate results derived from cohort and routine data.

## Aims and objectives

This study will examine the association between residence in a Communities First area and changes in mental health and social cohesion in a prospective controlled quasi-experimental, or ‘natural experiment’ design, set in a general adult population sample. Our primary objective is to determine:

1. What is the impact of the *Communities First* regeneration programme on mental health?

The interventions delivered as part of Communities First may also have an effect on levels of social cohesion. This association could occur through the positive individual and community effects of interventions on local friendship ties, collective attachment and rates of social participation.^17^ It is also clear that selective population migration needs to be taken into account in any examination of health inequalities over time,^18^ ^19^ and that the costs and benefits of interventions need to be assessed. Our secondary objectives therefore are to determine:

1. What is the impact of the *Communities First* regeneration programme on social cohesion?
2. To what extent does regeneration of a community result in population replacement rather than regeneration?
3. What is the impact of the Communities First programme on area-level inequalities in mental health and well-being, taking population migration into account?
4. To what extent can the benefits of the *Communities First* programme be considered to represent value for money?

## Methods and analysis

### Study design

The study will utilise intervention data and the eCATALyST study to create a prospective controlled quasi-experimental study—a ‘natural experiment’.^20^

### Setting

The study will be set in Caerphilly county borough, Wales, UK. Caerphilly borough has a population of around 178 000 with a large variation in levels of deprivation.^21^ Communities First intervention areas account for roughly one-third of the lower super output areas (LSOAs) in Caerphilly and one-quarter of the resident population.

### Participants

**Figure 1:**
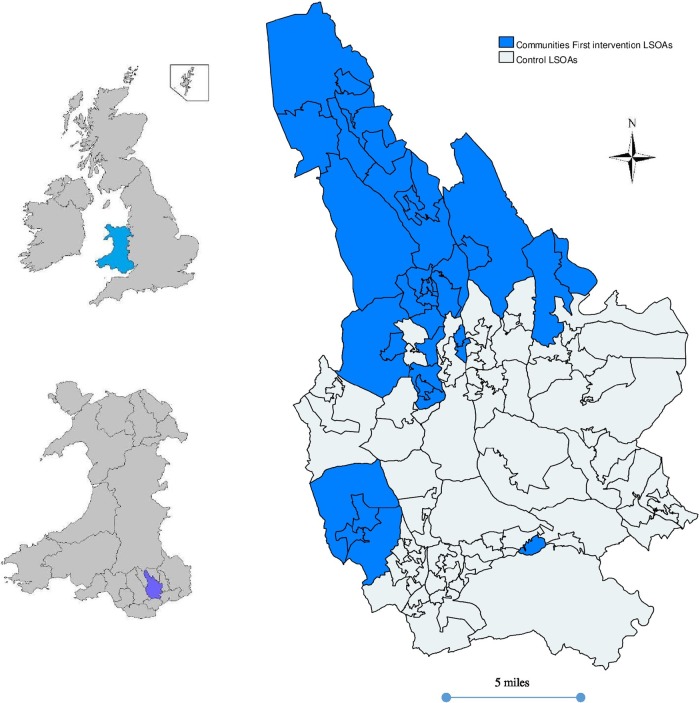
The 47 Communities First intervention lower super output areas (LSOAs) and 63 control LSOAs in Caerphilly County Borough (Wales, UK).

Data have been collected from 4426 participants aged 18–74 years on 31 May 2001 who responded to the 2001 and 2008 waves of the Caerphilly Health and Social Needs Electronic cohort study.^16^ The prospective controlled quasi-experimental study involves a comparison of the 1773 (40%) participants living in 47 LSOAs that received Communities First interventions between the two waves of data collection, with 2653 participants living in 63 LSOAs that did not receive any interventions (control areas; see figure 1).

### Use of existing record-linked datasets: the Secure Anonymous Information Linkage databank

The Secure Anonymous Information Linkage (SAIL) databank held within the Health Information Research Unit (HIRU) at Swansea University contains health, social and education data on three million residents of Wales, UK, and currently includes 13 data sets containing nearly one billion records.^22^ ^23^ Information governance for SAIL is overseen by an Information Governance Review Panel.^22^ The smallest geographical area for which data are already linked and may be released from the databank, after scrutiny for small numbers, is the 2001 Census LSOA.

The Welsh Demographic Service (WDS) data set held by NHS Wales Informatics Service (NWIS), the NHS organisation in Wales mandated to hold personally identifiable data, contains addresses for all individuals who register with a general practitioner (GP). Dates for each address record update are held, thereby providing durations of residency for several different homes and the ability to link to local environment exposures at each. This data set will be used to track population migration and record length of exposure in a Communities First area. The WDS contains address information linked anonymously at the individual level (the anonymised linking field, ALF) which is the primary key variable for record-linkage. Using a split-file technique, NWIS supplies ALFs for the whole population of Wales to the SAIL databank.^22^ ^23^

### Communities first intervention data

We will extract detailed data on each regeneration activity, including a text description, geographical location, duration and investment (£) using information held by Caerphilly county borough council. We propose to classify the interventions delivered as part of Communities First into seven domains of regeneration, informed by a scheme used to organise projects funded by the New Deal for Communities.^4^ Examples are provided below.

1. *Crime.* Reducing crime and the fear of crime through installing CCTV, street lighting and alarms;
2. *Education*. Providing educational support through after school/breakfast/holiday clubs, early learning and Sure Start;
3. *Health.* Projects to improve the health of residents directly through provision of sport equipment, staffing of sports clubs, health improvement interventions such as healthy eating projects;
4. *Housing and the physical environment.* Housing maintenance and repairs, environmental improvement such as redevelopment of waste land, maintenance of green space, parks, building of play grounds; building and maintenance of roads and cycle paths;
5. *Employment*. Improving employment prospects included providing advice to businesses; projects to develop of computer skills of the unemployed;
6. *Community*. Increasing social contact and participation including the building of community facilities, staffing of youth projects, funding of luncheon and widows clubs and community events;
7. *Infrastructure.* Spending on the running of the partnerships. This entailed spending on staff, stationary, and training.

The classification scheme may be amended following extraction of all information on interventions.

### The Caerphilly Health and Social Needs Electronic Cohort (eCATALyST)

The Caerphilly Health and Social Needs Electronic Cohort (eCATALyST) is a prospective cohort study residents of Caerphilly county borough, Wales, UK. The study has been described in detail elsewhere.^16^ Briefly, in 2001 a stratified random sample of 22 236 individuals aged 18 and above resulted in 10 892 respondents providing valid information on mental health. In 2008 the survey was repeated with 9551 participants who still resided in the borough. Of these, 4798 returned questionnaires, with 4426 providing data on their mental health at both waves. The study has detailed information on a wide range of social, demographic and economic risk factors (eg, age, gender, socioeconomic status, educational achievement, employment, household income, council tax band) health and lifestyle outcome data (eg, cardiovascular risk factors, SF-36v2 for the Mental Health Inventory (MHI) scale,^24^ limiting long-term illness), and perceptions of neighbourhood, including the Buckner Neighbourhood Cohesion scale.^25^

### Data from general practice on consultations and prescribing

The SAIL databank currently contains data on consultations and prescribing data for around 40% of the Welsh population. To date, data from 9 of the 29 GP practices in Caerphilly borough are record-linked in SAIL. This data will be used to compare information on common mental disorders from the eCATALyST data set to that defined using data from general practice. We have already defined a set of Read codes used by general practice to define a case of common mental disorder.^26^ Although only around one-third of the survey data set respondents will have linked GP data, this provides an opportunity to compare results for common mental disorders reported in the community survey to those that present to primary care.

### Primary outcome: mental health

Data on changes in mental health will be assessed using the 2001 and 2008 waves of eCATALyST. Mental health was assessed in 2001 and 2008 using the MHI (MHI-5) included in the SF-36 V.2 scale.^27^ ^28^ The validity and reliability of the MHI-5 are well established^28^ and the scores reflect the continuously distributed nature of mental health status in the population.^27^ ^28^ Respondents can achieve a total score within a range of 5–25, which can be transformed to a 0–100 scale.^29^ ^30^ The primary outcome measure for the analysis is change in mental health score, wave 2–wave 1, so that positive values indicate an improvement in mental health.

We will repeat our analysis using a set of Read codes used by general practice to represent the common mental disorders. The specific set of codes will build on work conducted by our group using data from general practice to define a common mental disorder.^26^ They will include codes for symptoms, diagnosis and treatments for psychiatric illnesses such as anxiety disorders and major depression.

### Secondary outcome measures

#### Social cohesion

Social cohesion was measured in both waves of eCATALyST study using a modified version of Buckner's Neighbourhood Cohesion Scale.^23^ Social cohesion was measured using eight items: ‘I visit my friends in their homes’, ‘The friendships and associations I have with other people in my neighbourhood mean a lot to me’, ‘If I need advice about something I could go to someone in my neighbourhood’, ‘I believe my neighbours would help in an emergency’, ‘I borrow things and exchange favours with my neighbours’, ‘I would be willing to work together with others on something to improve my neighbourhood’, ‘I rarely have a neighbour over to my house to visit’ (reverse scored), and ‘I regularly stop and talk with people in my neighbourhood’. We will derive small-area social cohesion scores using our ecometric methodology.^31^

### Mental health inequalities

The eCATALyST study includes the following measures of socioeconomic position at both waves^16^: Registrar General Social Class and the National Statistics Socioeconomic Classification (NS-SEC)^32^; housing tenure; council tax band of residence; full classification of employment status; and gross household income in two bands (above and below 60% of median income). Using the Welsh Index of Multiple Deprivation (WIMD) 2005 we will categorise each of the LSOAs into quintiles (based on four cut-points and equal counts of LSOAs) for aggregate analyses and use the WIMD score as a LSOA-level covariate in statistical models.

### Sample size

In this prospective controlled quasi-experimental study the sample size is fixed and so we can calculate the power available to detect a clinically important difference in our primary outcome measure of mental health, the Mental Health Inventory (MHI-5) scale scores between groups. Of the 4426 eligible survey participants, 1773 reside in 47 intervention LSOAs and 2653 in 63 control LSOAs. Comparing changes in the MHI-5 score between those living in regeneration areas and those living in control areas would have a power of 87% for detecting a difference of 2 in the mean score, allowing for clustering at LSOA-level. This is high power to detect a small, but clinically important, numerical difference in scores.

### Statistical analysis plan

The primary analysis will examine the association between changes in MHI-5 scores and residence in a Communities First intervention area or control area. Data on individuals nested within LSOAs will be available. Our analysis plan is:

1. Descriptive statistics for change in mental health, social cohesion and socioeconomic covariates 2001–2008, comparing residents of Communities First and control areas;
2. Multilevel linear models to examine changes in MHI-5 scores (2001–2008) and multilevel logistic models for the odds of a case of common mental disorder between residents of Communities First and control areas, adjusting for compositional characteristics including baseline age, gender and transitions in individual-level covariates recorded in eCATALyST. We will adjust for LSOA deprivation using quintiles of the 2005 Welsh Index of Multiple Deprivation (WIMD).^33^ We will then include interaction terms to examine whether the effect of Communities First varies according to individual and LSOA-level social cohesion. To investigate the different types of interventions, we will repeat models replacing the binary term for residence in a Communities First or control area with a categorical term for the different types of interventions.
  In order to address the secondary research questions:
3. We will fit further multilevel linear models described above to examine changes in levels of social cohesion;
4. We will assess the effect of population migration by including a term in these models for whether a participant has moved (yes/no) and whether that move was out of, or into, another Communities First LSOA. We will also compare a model, in which respondents are assumed not to have moved, with a model in which the correct migration is coded, following published methods.^19^
5. We will examine the effect of Communities First on mental health inequalities by modelling interactions between residence in a Communities First area and (1) baseline area deprivation Welsh Index of Multiple Deprivation (WIMD) 2005 scores^33^ and, (2) population subgroups (eg, gender, employment status);
6. A cost consequences analysis will be conducted and post-trial modelling employed to assess the cost consequences over longer time horizons than is possible within the study period. Changes in resources utilised over time in the Communities First areas relative to the control areas will be calculated and used in conjunction with the costs of setting up and delivering Communities First to generate the net cost of programme delivery per family; this will represent the incremental cost of providing the programme relative to usual service provision. The differences in primary, secondary and tertiary outcomes (including differences in utility scores derived from the SF-36 responses at each follow-up) will be used alongside the net cost of programme delivery to generate a set of indicators of relative cost-effectiveness within the study period, based on incremental cost and incremental outcomes.^34^ These will be used to provide indicators of the extent to which the programme can be viewed as representing value for money.

We plan to conduct a number of sensitivity analyses to examine: (1) duration of exposure to Communities First by repeating models with length of residence rather than a binary term for exposure; and, (2) contamination using definitions of each type of intervention according to their likelihood for contamination. We will also explore analysis using propensity scores in an attempt to promote balance across intervention and control areas. We will write these models in MLWiN,^35^ Stata,^36^ or R.^37^

### Ethics and dissemination

The study has been approved by the Information Governance Review Panel (IGRP) at Swansea University (Ref: 0266 CF) to link the Communities First intervention data to outcome data from the eCATALyST study, general practice records, as well as the WDS within SAIL. The IGRP reviews all applications to the SAIL databank and members include senior representatives from the British Medical Association (BMA), the National Research Ethics Service (NRES), Public Health Wales, NHS Wales Informatics Service (NWIS) and Involving People. NRES does not consider that studies using only anonymised data require its approval. The eCATALyST study received ethical approval for the baseline survey 2001 from the former Gwent Local Research Ethics Committee (REF: JW/CC/00/59(a)) and for the wave 2 survey in 2008, approved by the SE Wales Research Ethics Committee Panel C (ref 08/WSE03/25).

Findings will be disseminated through standard academic pathways including peer-review publications, presentations at national and international conferences, and updates on our study website (http://medicine.cardiff.ac.uk/clinical-study/communities-first-regeneration-programme/). In addition, we will present our findings to policy partners in Caerphilly county borough, Public Health Wales, the Welsh Government, as well as the partnership boards who currently deliver Communities First.

## Discussion

The proposed study is highly policy relevant. The Marmot Review of Health Inequalities has the creation of, “locally developed and evidence-based community regeneration programmes” as an objective to improve health and reduce health inequalities by 2020.^38^ This study exploits an opportunity to construct a natural experiment to evaluate the impact of a multimillion pound national regeneration programme on mental health. We will extract data on the location and type of interventions and nest it within an anonymously record-linked prospective cohort (eCATALyST) so that we will have detailed assessments on individual and household-level social, economic and health-based confounding factors which have been linked with changes in mental health. These detailed assessments are not commonly available in routine data sources which are typically used in prospective controlled quasi-experimental studies.^20^ ^39^ These individual-level confounding factors will be important in our planned analysis in the attempt to separate out the effects of Communities First, delivered on the basis of residence in a deprived area, from that area and individual-level socioeconomic disadvantage. Through linkage to routine data we can also examine effects on common mental disorders that present in primary care and provide a more sensitive assessment of exposure to the interventions funded by Communities First through information on length of residence in a Communities First area provided by the WDS.

## Supplementary Material

Author's manuscript

Reviewer comments
