# Supplementary material for: The Communities First (ComFi) study: protocol for a prospective controlled quasi-experimental study to evaluate the impact of area-wide regeneration on mental health and social cohesion in deprived communities
Source: BMJ Open. 2014 Oct 14;4(10):e006530. doi: 10.1136/bmjopen-2014-006530 (PMC4202000; doi:10.1136/bmjopen-2014-006530)
Supplement: Reviewer comments [file bmjopen-2014-006530.reviewer_comments.pdf]

PEER REVIEW HISTORY

BMJ Open publishes all reviews undertaken for accepted manuscripts. Reviewers are asked to complete a checklist review form (**http://bmjopen.bmj.com/site/about/resources/checklist.pdf**) and are provided with free text boxes to elaborate on their assessment. These free text comments are reproduced below.

ARTICLE DETAILS

**TITLE (PROVISIONAL)**	The Communities First (ComFi) study: protocol for a prospective controlled quasi-experimental study to evaluate the impact of area-wide regeneration on mental health and social cohesion in deprived communities
**AUTHORS**	White, James; Greene, Giles; Dunstan, FDJ; Rodgers, Sarah; Lyons, Ronan; Humphreys, Ioan; John, Ann; Webster, Chris; Palmer, Stephen; Elliot, Eva; Phillips, Ceri; Fone, David

This article was not externally reviewed at BMJ Open. Protocols that have been independently assessed prior to submission to BMJ Open are usually fast-tracked to publication on the grounds that further substantial changes will not be possible. This independent assessment will usually be external, independent review for both a substantial grant award from a non-commercial or government funder and ethics approval.
